# Supplementary material for: Longshot enables accurate variant calling in diploid genomes from single-molecule long read sequencing
Source: Nat Commun. 2019 Oct 11;10:4660. doi: 10.1038/s41467-019-12493-y (PMC6788989; doi:10.1038/s41467-019-12493-y)
Supplement: Supplementary file 1 — Supplementary Information [file 41467_2019_12493_MOESM1_ESM.pdf]

Supplementary Information for “Longshot enables accurate variant calling in diploid genomes from single-molecule long read sequencing”

Peter Edge and Vikas Bansal

This PDF file includes:

- Supplementary Methods
- Supplementary Figures 1-10
- Supplementary Tables 1-4
- Supplementary References

Supplementary Methods

Simulating a diploid genome

In order to simulate Illumina and PacBio reads, a diploid genome was generated from the hg19 reference genome (chromosomes 1-22). For simplicity, only SNVs were simulated. Heterozygous SNVs were placed uniformly at random at a rate of 0.001 and homozygous SNVs were placed uniformly at random at a rate of 0.0005. The SNV alleles were selected according to the genotype priors used by Li et al [1]. The phase for heterozygous genotypes was selected uniformly at random. Two fasta sequences for each chromosome were generated using the bcftools consensus command to insert the alleles at SNVs from each haplotype into the reference sequence.

Estimating coverage from aligned reads

For both Illumina and PacBio whole-genome read datasets, the median coverage for each dataset was measured by sampling 100,000 random positions from the genome using `bedtools random` [2] and counting the number of aligned reads covering each position (passing samtools flag filter `3844`). Then, the median value of the measured coverages was taken. For the simulated data, the median coverage was not measured, and instead the reported coverage is the read coverage that was generated for the simulation.

Identification of candidate SNVs

Longshot uses a simple (and standard) genotyping model for identifying candidate SNVs. For each position on the reference, the read bases piled up over that position are considered. The most frequent non-reference base is selected and denoted as the alt (1) allele. The three genotypes $G \in \{0/0, 0/1, 1/1\}$ are considered. Let $\mathbf{A}$ be the vector of allele observations over $\{0, 1\}$.

Using Bayes' rule,

$$p(G|\mathbf{A}) = \frac{p\,(\mathbf{A}|G)p(G))}{\sum_G p(\mathbf{A}|G)p(G)} \tag{1}$$

The probability of the observed pileup alleles is the product of their respective probabilities:

$$p(\mathbf{A}|G) = \prod_{a \in \mathbf{A}} (a|G) \tag{2}$$

$$p(a|G) = \begin{cases} 1-\varepsilon & \text{if } a = G_0 = G_1 \\ \varepsilon & \text{if } a \neq G_0 = G_1 \\ \frac{1}{2}(1-\varepsilon) + \frac{1}{2}\varepsilon & \text{otherwise} \end{cases} \tag{3}$$

ε is the probability of a sequencing error to a specific base. Since quality values are of limited use for SMS reads, we use our own estimated base-mismatch emission scores for this value (see the section 'alignment parameter estimation'). We can compute the probability of a non-reference genotype as:

$$p(0/1|A) + p(1/1|A) = 1 - p(0/0|\mathbf{A})$$

Finding non-repetitive anchors

For an SMS read overlapping a potential SNV site, non-repetitive anchor sequences to the left and right of the potential SNV site are identified to perform local realignment. For this, Longshot searches leftward (and rightward) from the SNV site to find a sequence of length k (default value 6) in the reference sequence where the aligned SMS read matches the reference exactly. This implies that the k-mer in the SMS read was likely sequenced from the template without error and is aligned correctly. This assumption may not hold when the reference sequence is repetitive. For example, consider a 6-mer AAAAAA that matches

between reference and SMS read perfectly. This may be a good anchor sequence if it is the only occurence of AAAAAA in the nearby reference sequence. It is a bad anchor if this is not the case; for instance, if it occurs inside an even larger homopolymer run of A's. We circumvent this issue by ignoring any potential anchor that occurs more than once on the reference sequence within the maximum anchor search window (100 bp by default). The rust-bio implementation of the BNDM algorithm is used to quickly perform this k-mer search[3, 4]. If the leftward or rightward anchor search exceeds half the size of the maximum anchor search window, then that position on the reference and read is used as the anchor regardless of any other factors. This means that in the worst case with default parameters, a realignment window of 100 bp will be formed around the potential SNV. In order to avoid forming realignment windows at a locus with large gaps, if a insertion/deletion/refskip event of length ≥ 20 is encountered near the window, the site is not realigned.

Pair-HMM realignment for clusters of SNVs

When multiple potential SNVs are located in close proximity, the realignment approach should consider the alternate haplotypes defined by these SNVs jointly rather than perform the local realignment for each SNV independently. This is especially important for false potential SNVs – it is common for a single true SNV to be misaligned in the original BAM so that the pileup-based scan identifies it as two or three potential heterozygous SNVs within a few bp of each other. Therefore, we use a simple approach to merge nearby SNVs into SNV clusters. For every pair of adjacent potential SNVs, we merge them into the same SNV cluster (and merge their realignment windows) if their realignment window boundaries overlap. For a cluster with n potential SNVs, we use the pair-HMM forward algorithm to realign against each of the 2^n possible short-haplotype sequences, which we will refer to as the set $\mathcal{H}$. For example, the possible haplotypes in the case of three potential SNVs are $\mathcal{H} = \{000, 001, 010, 100, 110, 101, 011, 111\}$ represented in bitstring form for the 3 SNV sites. We then use a Bayesian calculation similar to the single SNV case to calculate the probability of each possible short-haplotype $h \in \mathcal{H}$

$$p(h \mid \text{read}) = \frac{p(\text{read} \mid h)}{\sum_{h' \in \mathcal{H}} p(\text{read} \mid h')}$$

where $p(\text{read} \mid h)$ is calculated using the forward algorithm between the (multi-SNV) read-window and the haplotype sequence obtained by inserting into the reference sequence window each SNV in h. The short-haplotype h_{max} maximizing $p(h \mid \text{read})$ is selected and used to assign the call for each of the n alleles. The quality value q_i for each allele is calculated independently as:

$$q_i = \text{phred}\left(1 - \sum_{h_c} p(h_c \mid \text{read})\right)$$

where h_c is the set of haplotypes in $\mathcal{H}$ for which $h_c[i] = h_{\max}[i]$. In other words, it is 1 minus the sum of probabilities of all short haplotypes sharing the same best allele call in position i. The total computational complexity of all the realignments is $O(m^2 2^n)$, for read and haplotype windows of length m. For computational efficiency, we limit the cluster size (n) to a maximum value (default = 3) and break large clusters into smaller ones.

Priors on genotypes

Longshot uses the same approach as Li et al. [1] to estimate the prior probability of the genotypes. The prior probabilities for each SNV genotype (given the reference base) are derived assuming that heterozygous SNVs occur at a rate of 0.001 and homozygous SNVs occur at a rate of 0.0005. By default, Longshot differs from the Li et al approach in that a transition (Ts) mutation is assumed to occur at the same rate as a transversion (Tv) mutation. The prior probabilities can be specified as parameters to the software.

Haplotyping and measuring accuracy

Longshot produces a phased VCF as output, using the standard 'phase set' (PS) notation to delineate phased haplotype blocks. To assess the accuracy of the haplotypes assembled by Longshot, the output VCF was first filtered to remove SNVs with a low phase quality (PQ < 30). This value is similar to the genotype quality (GQ), except that it represents the confidence in the most likely phased genotype (0|0,0|1,1|0,1|1). The GQ, on the other hand, combines the heterozygous phased genotypes together to represent the most likely unphased genotype (0/0,0/1,1/1).

A switch error occurs when, at a single SNV, the phase (e.g. 0|1 or 1|0) of the assembled haplotype differs from the ground-truth haplotype with respect to the previously compared SNVs. A switch error indicates that the phase of the SNVs that follow will be different. If two consecutive switch errors occur such that the phase of only one SNV is incorrect, this corresponds to a 'mismatch' error or a short switch error. We calculated the switch and mismatch error rate separately and report a single error rate by adding these two error rates.

To compare the phasing performance of short reads with long reads using Longshot, we used HapCUT2 to assemble haplotypes using short reads and variants called using FreeBayes on the same set of reads [5]. Variants identified from short read WGS can also be paired with long read data to assemble long haplotypes [6]. This differs from Longshot, which requires no prior knowledge of SNVs (and genotypes) to assemble haplotypes. We compared the phasing accuracy of Longshot with this composite approach. For this, SNVs called using 30× Illumina WGS with the previously described filters were used with the extractHAIRS program to extract haplotype fragments in the PacBio reads mode (`--pacbio 1`). Then, the fragments were used to assemble haplotypes with HapCUT2 (`v1.1`). The resulting haplotypes were filtered for a minimum mismatch quality (similar to the phase quality described for Longshot) of 30.

Separation of reads by haplotype

Let $H = (H_1, H_2)$ be the final pair of haplotypes output by Longshot. Assuming that the prior probability of a read originating from H_1 or H_2 is equal, the probability that a read r was sampled from haplotype H_1 can be calculated as:

$$\frac{p(r|H_1)}{p(r|H_1) + p(r|H_2)}$$

The read is assigned to H_1 if this probability is at least T (default value = 0.99), assigned to H_2 if the probability is $\leq 1 - T$ and left unassigned otherwise.

Alignment Parameter Estimation

In order to estimate allele probabilities using the pair-HMM, it is necessary to know the best alignment parameters for SMS reads. Specifically, the parameters are transition probabilities between every state in {MATCH, INSERTION, DELETION} (with outgoing probabilities for a state summing to 1) as well as emission probabilities for a pair of aligned bases. We use a single emission probability for matched bases, and a single emission probability for mismatched bases. In order to use parameters that accurately reflect the data, we estimate these probabilities directly from the alignments in the bam file. While the bam alignments are too inaccurate for sensitive genotyping, we can expect the alignments to roughly reflect the probabilities of insertion, deletion, and base mismatch errors for the reads. This approach also assumes that variants from the reference genome are significantly less common than read errors, which is true for SMS reads from the human genome. We perform a single scan over every CIGAR string and sequence in the BAM, and transition between MATCH, MISMATCH, and DELETION states according to the CIGAR string. The number of observed transitions from each state to itself or other states is counted and converted into probabilities by dividing by the total transitions out of the outgoing state. The aligned bases at each step in the CIGAR are tracked, and the total number of matching and mismatching bases are used to estimate the emission probability for matched vs. mismatched bases.

Variant calling using Clairvoyante and WhatsHap

We installed Clairvoyante 1.02 using the Bioconda method described on the github (`https://github.com/aquaskyline/Clairvoyante`), but encountered a runtime error related to CPU affinity alteration that we avoided with a small fix to the code, described in this github issue (`https://github.com/aquaskyline/Clairvoyante/issues/27`). We used the pre-trained models available at `http://www.bio8.cs.hku.hk/trainedModels.tbz` that were trained at learning rate 1e-3 for 999 epochs. We used the model trained on NA24385 to call variants on NA12878 and the model trained on NA12878 for other genomes. We ran Clairvoyante using commands of the form:

```
clairvoyante.py callVarBam --chkpnt_fn {model} --ref_fn ref.fa \
--bam_fn ngmlr_alignments.bam --ctgName {chrom} \
--call_fn {chrom}.out.vcf --sampleName {sample_name} \
--threshold 0.2 --minCoverage 4 --threads 4
```

Comparison of precision and recall values between methods requires choosing a threshold for the variant quality. For Clairvoyante, following the authors' approach [7], we used the 0.2 allele frequency cutoff and the variant quality threshold that maximized the F1-score.

We used WhatsHap 0.18 (https://bitbucket.org/whatshap/whatshap) using the potential variants discovered in step 1 of the Longshot algorithm as input. The program was run with the following command for each genome:

```
whatshap genotype --ignore-read-groups --reference ref.fa \
-o {chrom}.out.vcf potential_snvs_{chrom}.vcf input.bam
```

After calling variants, the VCF was filtered using the same maximum coverage filter as Longshot. Both methods were run separately on each chromosome on an Intel Xeon CPU E5-2670 0 @ 2.60GHz.

Variant calling using Nanopolish

We used Nanopolish version 0.11.1 to call variants using the rel6 version of the NA12878 ONT data (https://github.com/nanopore-wgs-consortium/NA12878/blob/master/Genome.md). The raw fastq files were downloaded and aligned with minimap2 to hg38 reference genome. First, we downloaded the individual fast5 files and ran nanopolish index with command of the form:

```
nanopolish index \
-d fast5/Bham/FAB39043-3709921973_Multi \
.... \
-d fast5/Bham/FAB41174-3976885577_Multi \
-s fast5/rel_6_sequencing_summary.txt rel_6.fastq.gz
```

For variant calling, we divided chromosome 20 into chunks of size 1 MB and ran nanopolish on the chunks using commands of the form:

```
nanopolish variants --threads 4 --ploidy 2 -q cpg --window chr20:{start}-{end} --reads rel_6.
    fastq.gz --bam minimap2_alignments.bam --genome hg38.fa --outfile chr20.{start}.{end}.vcf
```

After this, the VCF's from the individual chunks were recombined. We note that we ran nanopolish in the methylation aware mode since it yielded better results.

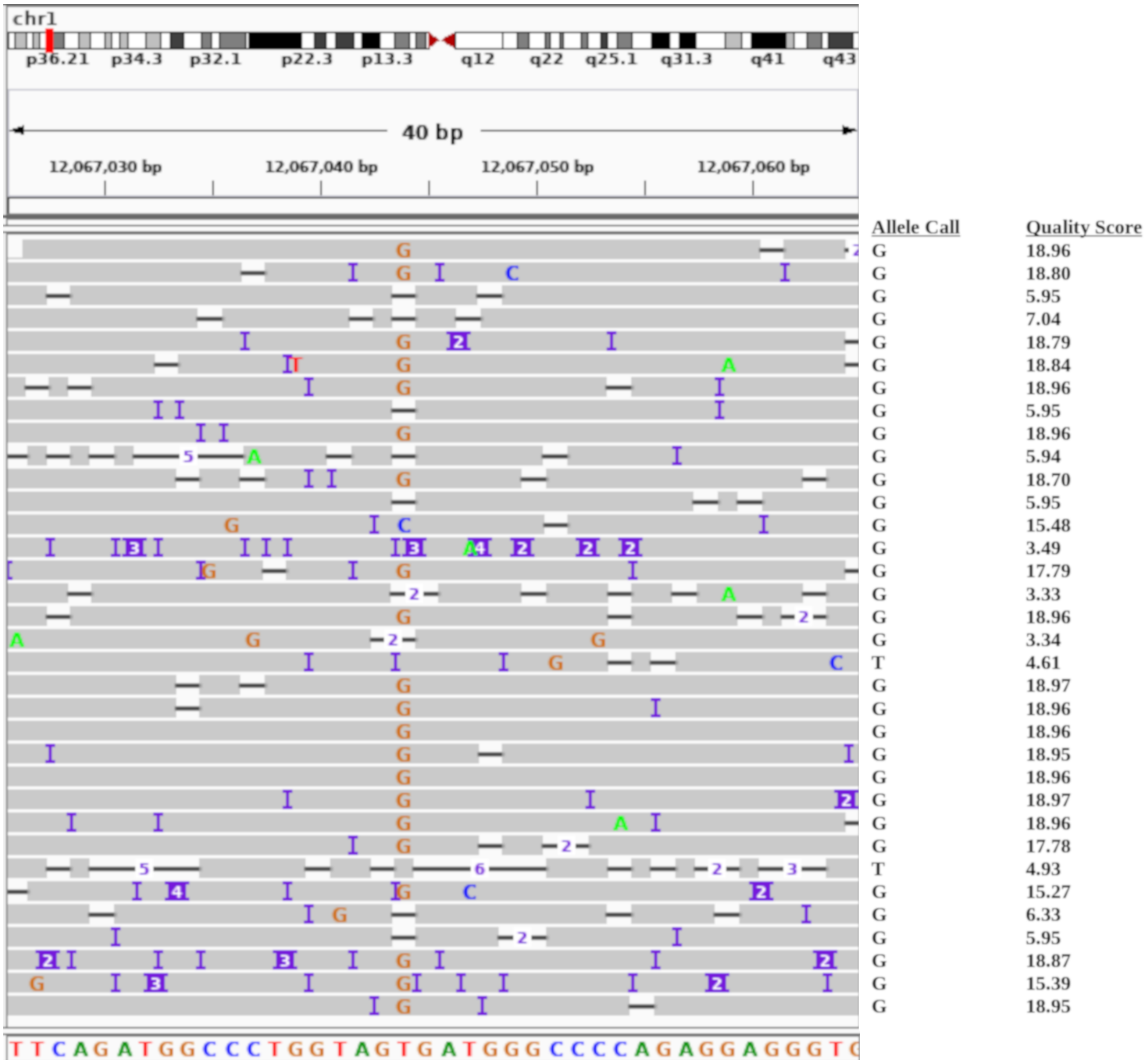

Supplementary Figure 1: Illustration of reference bias in SMS read alignments. PacBio SMS reads covering a homozygous SNV (chr1:12,067,044 T→G) site in the individual NA12878 are shown (visualized using IGV). Frequent indel errors in the SMS reads, combined with a bias in the alignment algorithm to favor the reference allele, results in 3 reads that contain the reference allele at the SNV site, 1 read containing a C, and 9 reads that contain a deletion. As a result, the variant can incorrectly be called as a heterozygous SNV. Realigning each read to both the reference allele and the alternate allele and selecting the most likely alignment can ameliorate this bias. To the right of each read, the most likely allele call and quality value calculated by Longshot using the Pair-HMM realignment strategy is shown. All reads, except two reads with very low quality values (< 5), support the 'G' allele resulting in the correct homozygous SNV call.

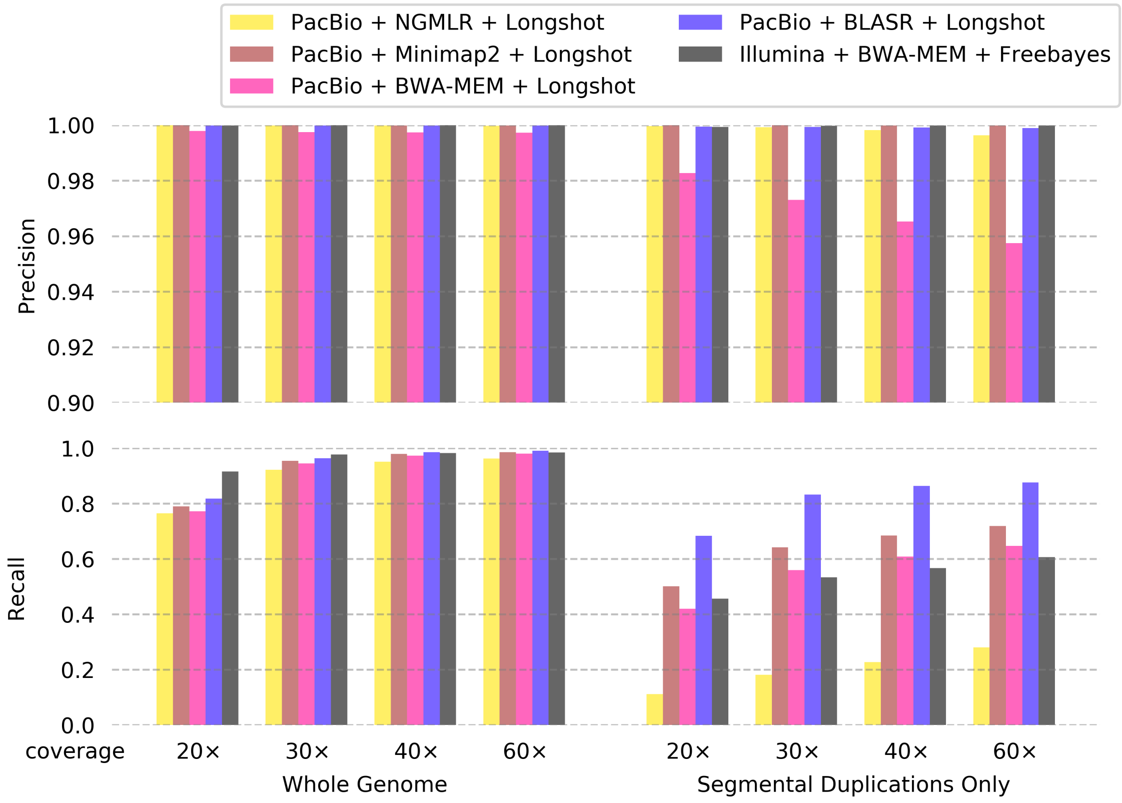

Supplementary Figure 2: Comparison of precision and recall of SNV calling using different long-read mapping tools. Simulated Illumina and PacBio reads were generated at multiple coverages (20×, 30×, 40× and 60×) from chromosomes 1-22 (hs37d5 reference genome) and variants were called using either Longshot (PacBio) or FreeBayes (Illumina). SMS reads were mapped with multiple mapping tools (NGMLR, BWA-MEM, MINIMAP2, and BLASR) for comparison. Precision **(top)** and Recall **(bottom)** of called variants were assessed across the entire chromosome **(left)** and within segmental duplications with 95% or greater sequence identity **(right)**.

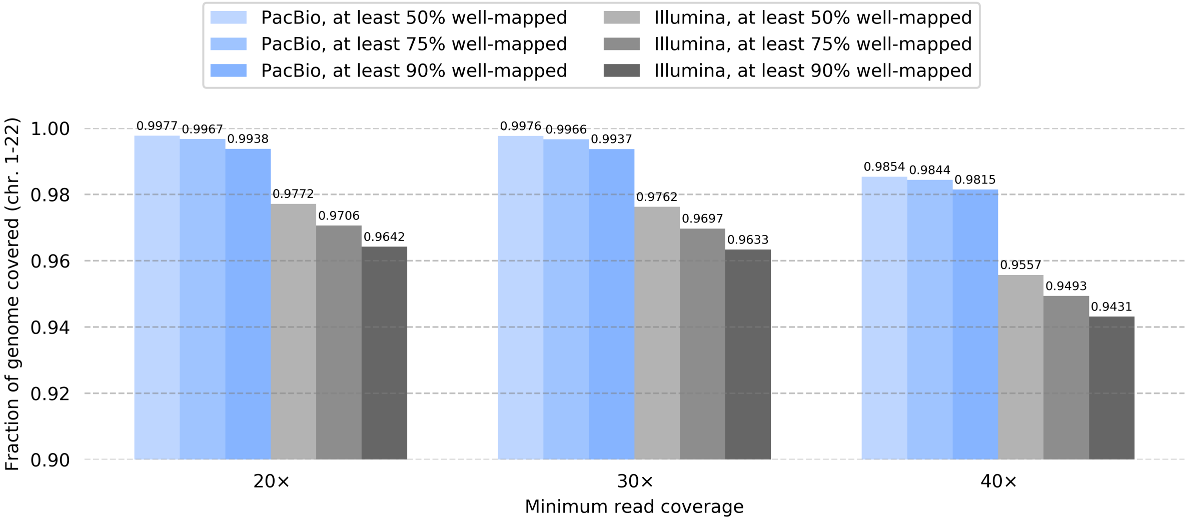

Supplementary Figure 3: Comparison of the mappability of short reads with long reads using simulated data. Illumina short reads and PacBio SMS long reads were each simulated at 60× coverage and mapped to the genome with BWA-MEM and BLASR, respectively. For every position in the genome, the coverage of primary read mappings was assessed. The positions in the genome were filtered for those with at least 20×,30×, and 40× coverage of primary read mappings. Of those positions, it was determined what fraction of the mappings were "well-mapped", or passing standard filters and having MAPQ $\geq$ 30. The number of positions meeting the minimum coverage cutoff and also meeting a minimum "well-mapped read" cutoff of at least 50%, 75%, 90% are shown as a fraction of total genomic positions (excluding "N" positions).

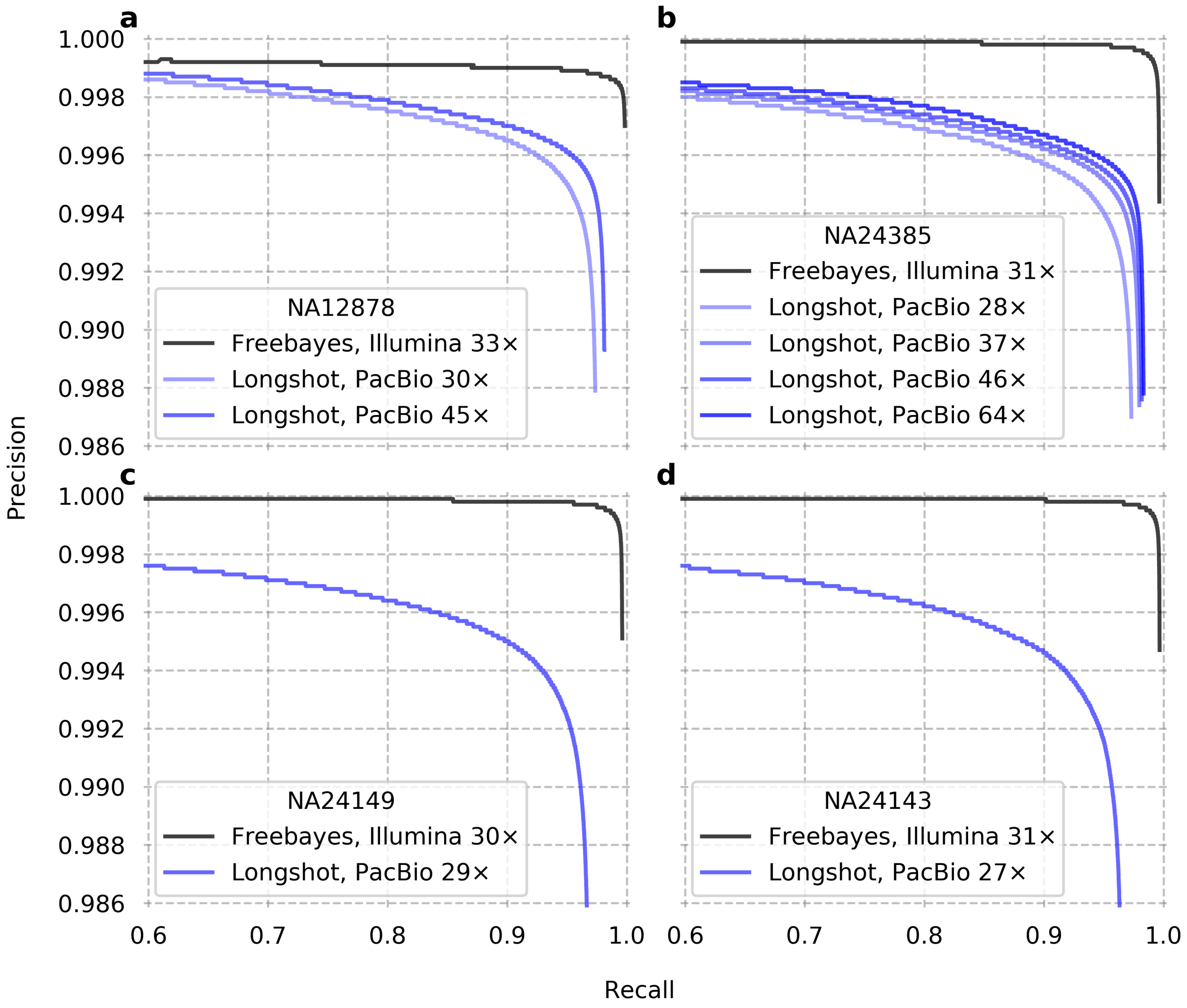

Supplementary Figure 4: Precision-Recall curve for SNV calling on four individuals: (a) NA12878, (b) NA24385, (c) NA24149, and (d) NA24143. For each individual, variants were called from Illumina short reads using FreeBayes and from whole-genome PacBio SMS reads using Longshot. Precision and recall were calculated using GIAB SNV calls within high-confidence regions. Points on each curve are obtained by varying the minimum Genotype Quality (GQ) cutoff.

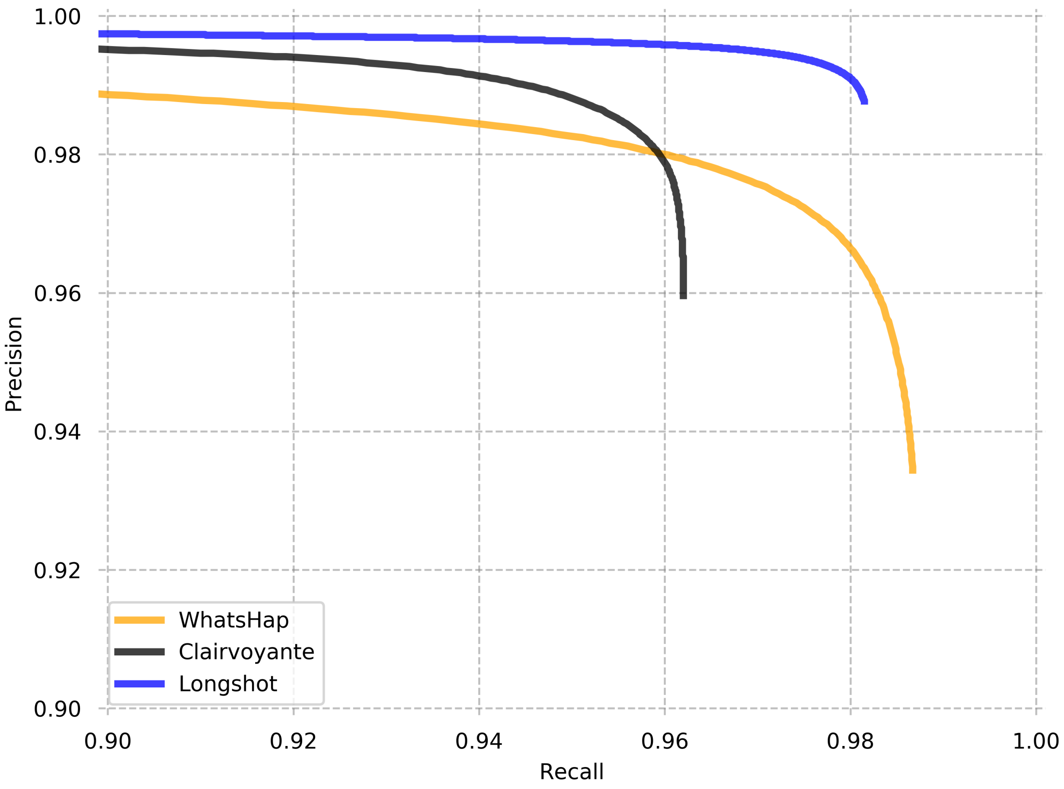

Supplementary Figure 5: Comparison of precision and recall of SNV calling using different variant calling methods (Longshot, Clairvoyante and WhatsHap), on the NA12878 PacBio dataset. Reads aligned using the NGMLR tool were used for variant calling using each method. Precision and recall were calculated using GIAB SNV calls within high-confidence regions.

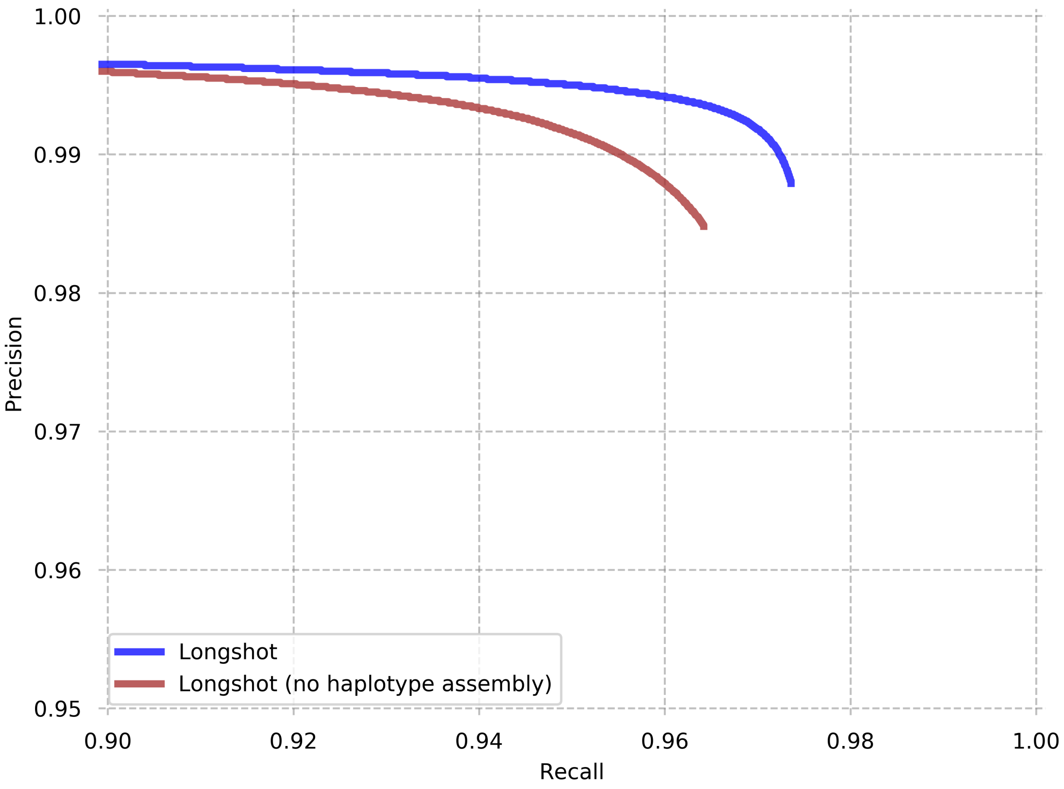

Supplementary Figure 6: Precision-Recall Curves for Longshot with and without phased genotyping. Longshot was run as normal on the whole-genome NA12878 PacBio data (downsampled to 30× coverage), as well as with "no haplotype assembly" (skipping step 3 of the algorithm).

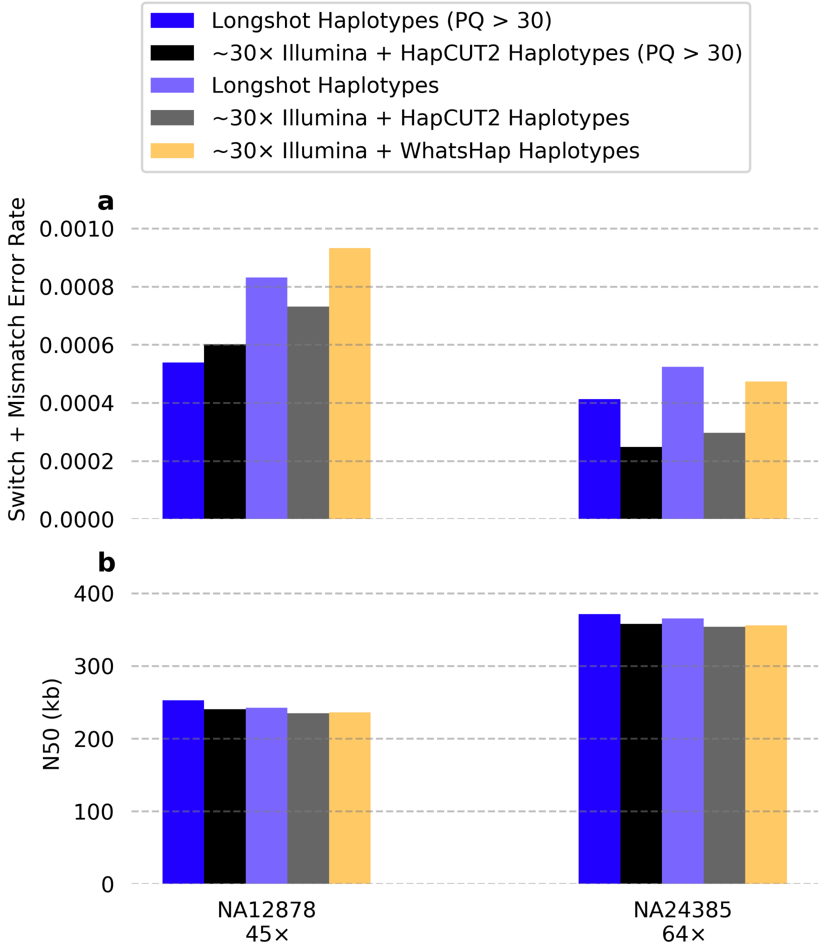

Supplementary Figure 7: Comparison of the accuracy of haplotypes assembled with Longshot, HapCUT2 and WhatsHap for two genomes: NA12878 (45×) and NA24385 (64×). For running HapCUT2 and WhatsHap, variants identified from ∼30× coverage Illumina sequencing were used as input for phasing. The accuracy of the resulting haplotypes **(a)** was measured using the combined switch error rate (long switches and mismatches), and the completeness **(b)** was measured using the N50 length of the haplotypes. Results are also shown for Longshot and HapCUT2 after filtering for SNVs with Phase Quality (PQ) greater than 30.

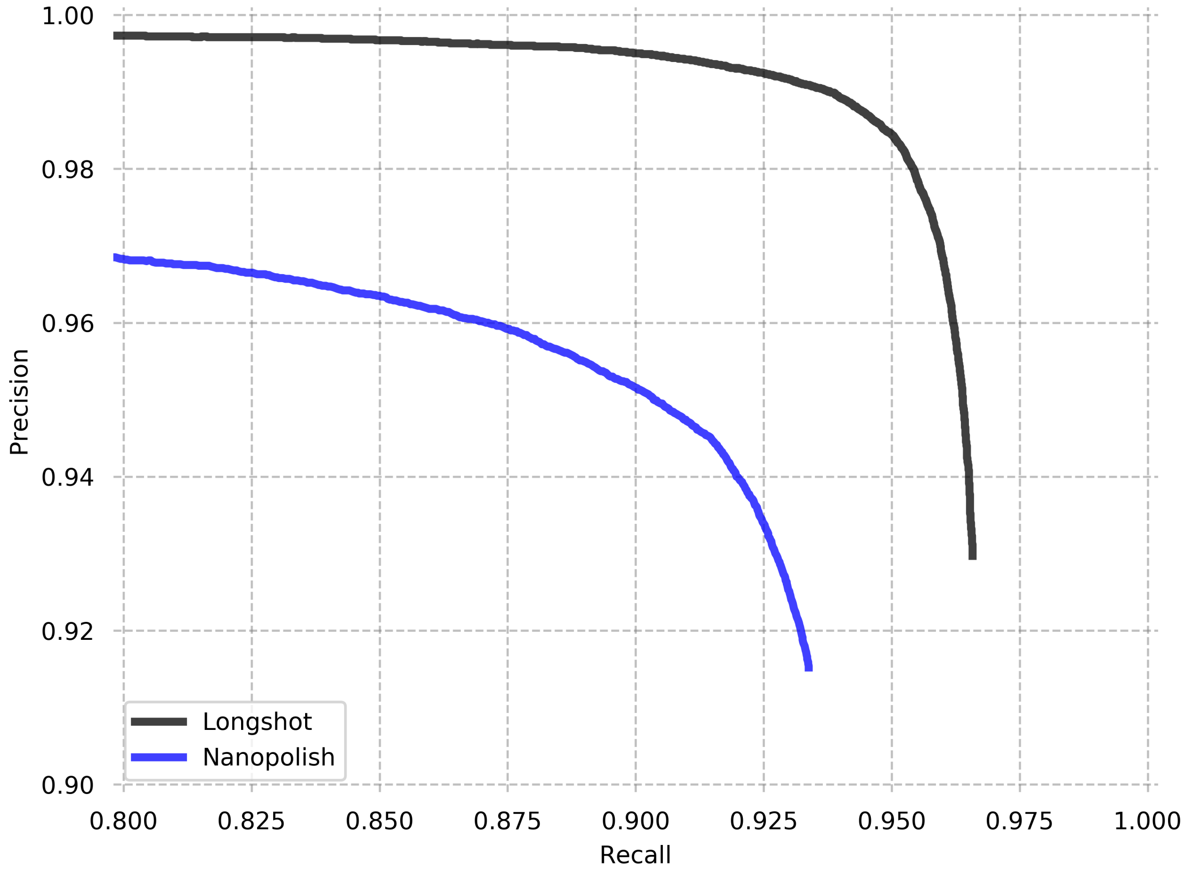

Supplementary Figure 8: Precision-Recall curve for SNV calling using whole-genome Oxford Nanopore data for NA12878 ($\sim$ 37$\times$ coverage). Precision and recall were calculated using GIAB SNV calls within high-confidence regions of the genome. Points on the curve were obtained by varying the cutoff of the Genotype Quality (GQ) score for Longshot and the QUAL score for Nanopolish. Results shown are based on chromosome 20 only.

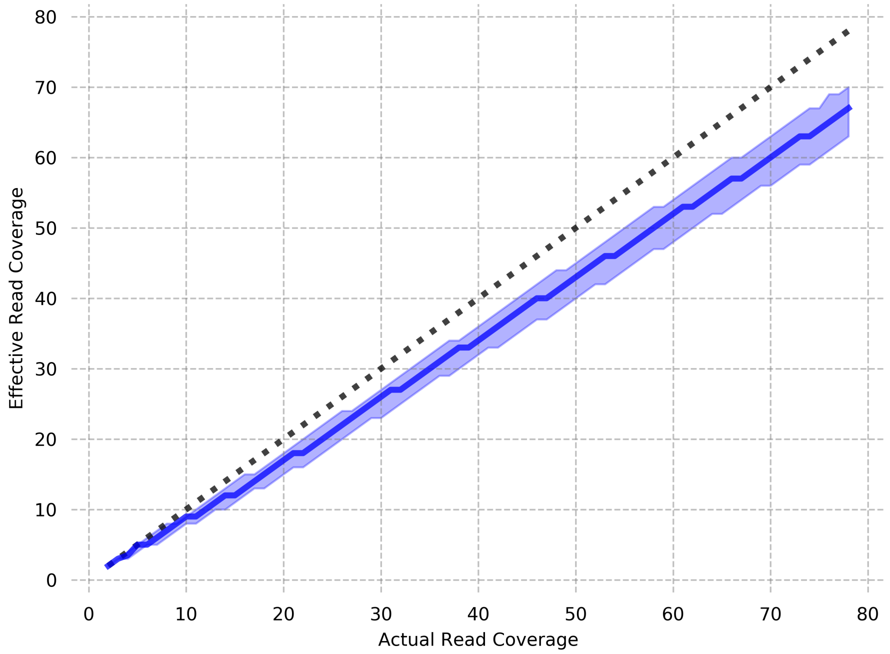

Supplementary Figure 9: Actual vs effective read coverage in PacBio SMS data. At each SNV site, alleles for which the quality value estimated by the allelotyping method in Longshot is lower than a threshold (default = 7) are discarded; this reduces the effective read coverage. The data shown here is for SNV sites from the 45× coverage NA12878 PacBio dataset (chromosome 1 only). The median effective read coverage (as well as 1st and 3rd quartiles) is plotted for variant sites with the same actual read coverage. Source data is provided as a Source Data file.

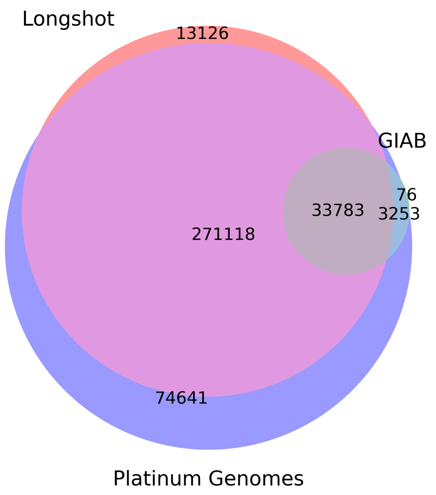

Supplementary Figure 10: Comparison of Platinum Genomes variant calls (outside GIAB confident regions) with Longshot variants for NA12878. Variants were called on chr1-22 with Longshot using 45× coverage PacBio SMS reads for NA12878. The Longshot variants, GIAB variants, and Platinum Genomes variants were each filtered for regions that are outside GIAB confident regions, but inside the Platinum Genome called regions. 79.6% of SNVs in these difficult-to-call regions are shared between the Platinum Genomes calls and the Longshot calls.

Genome	Read Coverage	SNVs called	Precision	Recall	SNVs outside GIAB-HC regions	Run time (hours)	Memory (mean, GB)	Memory (max, GB)
NA12878	30×	3518530	0.994	0.959	515870	27:31	3.23	4.99
NA12878	45×	3567475	0.995	0.973	520375	36:41	3.71	5.76
NA24385	28×	3585223	0.993	0.961	660938	38:30	3.26	5.08
NA24385	37×	3628953	0.993	0.973	666769	47:03	3.66	5.73
NA24385	46×	3642443	0.993	0.977	666733	68:40	3.87	5.71
NA24385	64×	3651477	0.994	0.980	666447	92:48	4.56	7.00
NA24149	29×	3529531	0.992	0.952	776106	37:32	3.25	4.76
NA24143	27×	3528836	0.992	0.945	772344	35:22	3.16	4.66

Supplementary Table 1: Summary of SNVs called using Longshot on whole-genome PacBio SMS data for multiple individuals. Only variants called on the autosomes (chromosome 1-22) are reported. For the NA24385 and NA12878 individuals, variants were called at multiple levels of coverage by downsampling. Precision and recall were calculated inside GIAB high-confidence (GIAB-HC)) regions using the GIAB variant set for each individual as the ground truth. Longshot was run separately on each chromosome each using a single CPU core of an Intel Xeon CPU E5-2670 0 @ 2.60GHz. The run-time is the total measured walltime to process all chromosomes (sum of all individual chromosome walltimes). The mean memory (averaged across chromosomes), and also the maximum memory usage (across chromosomes) are also provided.

Genome	False Positives (FP)	FP Enrichment	False Negatives (FN)	FN Enrichment
Misgenotyped SNV	0.050	-	0.014	-
Near Indel	0.714	176.99	0.053	13.17
In homopolymer	0.323	5.70	0.195	3.44
In homopolymer but not near indel	0.025	0.46	0.163	2.94
In STR	0.008	27.90	0.002	7.20
In LINE	0.286	1.32	0.215	0.99
In SINE	0.122	0.81	0.221	1.46

Supplementary Table 2: Fractions of False Positive (FP) and False Negative (FN) variant calls that were misgenotyped or coincide with genomic features. Variants were called using Longshot on chr1 using 45× coverage SMS reads for NA12878. The FP and FN variants were determined with respect to the ground truth (GIAB variant set within GIAB confident regions). In order to reason about why the FPs and FNs occurred, the fraction of those variants that fall into different categories was counted. For comparison, 100,000 random positions from chr1 were selected, filtered by the GIAB confident regions and subjected to the same analysis. The fold enrichment compared to the random positions is shown as a measure of the significance.

Genome	Read Coverage	Precision (known indels not filtered)	Precision (known indels filtered out)	Recall (known indels not filtered)	Recall (known indels filtered out)
NA12878	45×	0.995	0.997	0.974	0.969
NA24385	64×	0.994	0.996	0.979	0.974
NA24149	29×	0.993	0.995	0.948	0.943
NA24143	27×	0.993	0.995	0.941	0.936

Supplementary Table 3: Improvement in variant precision by filtering out SNVs near known indel variants. Most false positive (FP) variants called with Longshot occur at true indel variant sites that are mistaken as SNVs. Filtering out SNVs occuring within 5 bp of known indel sites (Mills + 1000G Indel variant set from the GATK bundle) results in improved precision at the cost of a small reduction in recall.

Genome	Technology	Method	Precision	Recall
NA12878	PacBio	Longshot	0.9947	0.9701
NA12878	PacBio	DeepVariant	0.9819	0.9739
NA12878	ONT	MarginPhase	0.809	0.769
NA12878	ONT	Clairvoyante	0.9148	0.7518

Supplementary Table 4: SNV calling accuracy for different methods on PacBio and Oxford Nanopore data for NA12878. The precision and recall values for DeepVariant on the PacBio data were obtained from Poplin et al. [8] and based on three chromosomes (20, 21 and 22). For a direct comparison, we calculated the precision and recall for Longshot using the BLASR-aligned bams on these three chromosomes only. The precision/recall for Clairvoyante was obtained from Supplementary Data (Luo et al. [7]) and was based on rel3 release of the ONT data aligned with NGMLR. The accuracy values for MarginPhase were obtained from [9].

Supplementary References

[1] Li, R. *et al.* SNP detection for massively parallel whole-genome resequencing. *Genome Res.* **19**, 1124–1132 (2009).

[2] Quinlan, A. R. & Hall, I. M. Bedtools: a flexible suite of utilities for comparing genomic features. *Bioinformatics* **26**, 841–842 (2010).

[3] Köster, J. Rust-bio: a fast and safe bioinformatics library. *Bioinformatics* **32**, 444–446 (2015).

[4] Navarro, G. & Raffinot, M. A bit-parallel approach to suffix automata: Fast extended string matching. In *Annual Symposium on Combinatorial Pattern Matching*, 14–33. Springer (1998).

[5] Garrison, E. & Marth, G. Haplotype-based variant detection from short-read sequencing preprint at https://arxiv.org/abs/1207.3907 (2012).

[6] Edge, P., Bafna, V. & Bansal, V. Hapcut2: robust and accurate haplotype assembly for diverse sequencing technologies. *Genome Res.* **27**, 801–812 (2017).

[7] Luo, R., Sedlazeck, F. J., Lam, T.-W. & Schatz, M. C. A multi-task convolutional deep neural network for variant calling in single molecule sequencing. *Nat Commun* **10**, 998 (2019).

[8] Poplin, R. *et al.* A universal SNP and small-indel variant caller using deep neural networks. *Nat. Biotechnol.* **36**, 983–987 (2018).

[9] Ebler, J., Haukness, M., Pesout, T., Marschall, T. & Paten, B. Haplotype-aware diplotyping from noisy long reads. *Genome Biol.* **20**, 116 (2019).
